# A Comparison between Shear Bond Strength of VMK Master Porcelain with Three Base-metal Alloys (Ni-cr-T3, VeraBond, Super Cast) and One Noble Alloy (X-33) in Metal-ceramic Restorations

**Published:** 2013-12

**Authors:** A Ahmadzadeh, A Neshati, N Mousavi, S Epakchi, F Dabaghi Tabriz, AH Sarbazi

**Affiliations:** a Dept. of Prosthodontics, School of Dentistry, Ahwaz University of Medical Sciences, Ahwaz, Iran; b Resident of Prosthodontics, School of Dentistry, Ahwaz University of Medical Sciences, Ahwaz, Iran; c Resident of Operative Dentistry, School of Dentistry, Ahwaz University of Medical Sciences, Ahwaz, Iran; d Prosthodontic Specialist

**Keywords:** Shear bond, Base-metal Alloy, Noble Alloy, Porcelain

## Abstract

**Statement of Problem:** The increase in the use of metal-ceramic restorations and a high prevalence of porcelain chipping entails introducing an alloy which is more compatible with porcelain and causes a stronger bond between the two. This study is to compare shear bond strength of three base-metal alloys and one noble alloy with the commonly used VMK Master Porcelain.

**Materials and Method: **Three different groups of base-metal alloys (Ni-cr-T3, Super Cast, and VeraBond) and one group of noble alloy (X-33) were selected. Each group consisted of 15 alloy samples. All groups went through the casting process and change from wax pattern into metal disks. The VMK Master Porcelain was then fired on each group. All the specimens were put in the UTM; a shear force was loaded until a fracture occurred and the fracture force was consequently recorded. The data were analyzed by SPSS Version 16 and One-Way ANOVA was run to compare the shear strength between the groups. Furthermore, the groups were compared two-by-two by adopting Tukey test.

**Results:** The findings of this study revealed shear bond strength of Ni-Cr-T3 alloy was higher than the three other alloys (94 MPa or 330 N). Super Cast alloy had the second greatest shear bond strength (80. 87Mpa or 283.87 N). Both VeraBond (69.66 MPa or 245 N) and x-33 alloys (66.53 MPa or 234 N) took the third place.

**Conclusion:** Ni-Cr-T3 with VMK Master Porcelain has the greatest shear bond strength. Therefore, employment of this low-cost alloy is recommended in metal-ceramic restorations.

## Introduction

Metal-ceramic restorations are broadly used in dental restoration procedures. These restorations consist of a cast metal substructure or coping which is coated with ceramic [1]. Initially, gold alloys were used to make these restorations but with a rise in gold prices since 1960, the application of lower-cost alloys has been more common. 

In the same thickness, base-metal alloys are more rigid than noble-alloys and this mechanical property is considered as an advantage in their applications [2]. Particular disadvantages of base-metal alloys include harmful biologic effects and uncontrolled formation of oxide layer [3-4]. Ni-Cr alloys can be regarded as an adequate substitute for noble alloys. Ni-Cr alloys have a higher modulus of elasticity and they are more capable of withstanding loads without much bending. They have superior strength in reduced thickness, and consequently, they provide much more space for porcelain and improve the esthetic qualities [5]. Furthermore, because of their high thermal compatibility with porcelain and their low cost, they are popular among clinicians and technicians [6].The clinical studies, with increasing reports of porcelain chipping, have aroused great worries. Porcelain chipping affects the function and the esthetic of restorations but a strong bond and an adequate adjustment of the applied alloy and porcelain can play a significant role in preventing the occurrence of this predicament [7]. According to the previous studies; after corrosion the porcelain chipping is the next common cause of treatment failure in the metal-ceramic restorations [8]. Porcelain chipping can be caused by trauma, partial occlusal adjustment, parafunctional habits, flexural fatigue, CTE (coefficients of thermal expansion), mismatching of the porcelain and poor design of the metal framework, and flaws in the laboratory processes [9]. Some factors such as van der Waals forces, mechanical interlocking, compressive bonding force, chemical bonding between porcelain and the oxide layer on the surface of metal, the roughness of the metal surface, and wetting properties would affect the bond strength of porcelain and alloy [9-10]. It is worth mentioning that many studies have conveyed chemical bonding as the first important factor that affects the bond strength between metal and porcelain. 

The elements in gold alloys, such as iron, tin, and indium and the elements in base metal alloys, such as chrome and beryllium contribute to the formation of oxide layer on the surface of metal [11]. Of course these elements are present in a layer several millimeters away from the metal-porcelain interface and promote the formation of a strong metal-porcelain bond. Hence, the concentration of these elements in alloys and their compatibility with the other elements are determining [8, 10, 12]. These elements should be compatible with the employing porcelain so that the bond between the two materials would have enough strength [2]. The porosities in the alloy surface can affect the bond strength. Palladium (Pd), for instance, increases the porosities in the noble alloys and in case of poor wetting of porcelain, this element expresses a negative effect and reduces the bond between porcelain and metal [4, 13]. Dental porcelains are strong enough under compressive and tensile loads; whereas they are weak under sheer loads [8-9]. To increase the bond strength between the metal and porcelain and to reduce the fracture risks under shear loadings, beryllium is added to the base-metal alloys. Beryllium (Be) reduces the melting point; facilitates casting; increases the fluidity and controls the oxide layer. A low concentration of beryllium (less than 2%) improves the bond strength and a conversely, the high concentrations form an excessively thick oxide layer and eventually reduce the bond strength between metal and porcelain. Regarding the properties of beryllium, dental laboratories add this element to the base-metal alloys [1, 5]. CTE is another important factor which influences the bond strength between metal and porcelain. Concerning the generated residual compressive forces in the porcelain upon cooling and to improve the bond strength between metal and ceramic; the value of CTE of the metal should be approximately 0.5×10^-6^ K^-1^ higher than that of the porcelain [9].

The strength of the framework does not depend solely on the employed alloy but also on the design used [2]. Bu-Kyung et al. pointed out that shear bond strength of veneering porcelain to metal cores, in noble and base-metal alloys, are not different [16].

The development in the system of color determination and the importance of esthetic issues, and the introduction of 3D Master Porcelain system has led to a better compatibility between color and appearance in metal-ceramic restorations [14]. 

Due to the high prevalence of porcelain chipping and because of the importance of shear bond strength between metal and porcelain and in search of an alloy with the maximum compatibility with widely-used porcelain, VMK Master Porcelain powder; the current study was planned. This study aimed to compare the shear bond strength of VMK Master Porcelain fused to one type of noble alloys, as the control group, and the available and conventional base-metal alloys In Iranian market.

## Materials and Method

To conduct this study, three types of metal-base alloys were selected as follows: VeraBond (Aalba dent Inc.; Cordelia, C.A, USA), Super cast (Talladium Inc.; Valencia, USA), and T3 (TiconiumInc; USA) and one type of noble alloys (Nourafranco; Switzerland- Italy Inc., Italy). For each type of alloy, 15 specimens of wax disks (0.4 mm in thickness and 10 mm in diameter) were waxed up and the diameter was measured by a digital caliper (Mitutoyo; Japan). Concerning the melting point of the alloys, a phosphate-bonded investment (Termo Cast; Polidental Ltd, Sao Paulo, SP, Brazil) was adopted. Phosphate lining were being loosen after 45 minutes, hence, the researchers placed the molds in the furnace (Onmad; Filli Manifred, Torino, Italy). After casting, the molds were cooled down at room temperature. After un-molding, the investment residues were removed through sandblasting with 50 μm Al2o3 particles with a Trijet machine (Labor dental; São Paulo, Brazil). The finishing process of the specimens was accomplished by the laboratory tungsten bur (H79 NEF, 104, 23; Brasseler USA, Savannah, GA) and a low-speed handpiece (KavoEwl, type 4005; Leutkrichim, Algau, Germany) with a speed of 10000 rpm to eliminate the casting defects. The thickness of the specimens was adjusted again using a digital caliper. Then the specimens were examined under a surgical microscope (Carl Zeiss; Germany) at 25 X magnifications to ensure the casting process has caused no defect, and then the specimens with casting problems were eliminated. The remained specimens were then sandblasted with 50 μm Aluminum oxide particles. Then, they were placed in an ultrasonic machine (Mini Sono Cleaner CA 1470; KaijoDenki Co. Ltd., Tokyo, Japan) for 10 minutes in order to eliminate any probable surface contamination. Oxidation was performed in air at 980º C temperature for 10 minutes. Porcelain build-up followed two sequences: one for the opaque layer and the other for the dentin layer. The build-up of the opaque layer of VMK Master Porcelain (Vita; Zehnfabrik, Bad Sackingen, Germany) was done according to the manufacturer’s instruction and then it was put in the furnace (Vita Vacumot 40T; vita Zehfabrik, Germany) for the first firing. Then the initial finishing was done with diamond burs and after carrying out an evaluation by a digital caliper (Mitutoya; Japan), the researchers built up the dentin porcelain in the required thickness according to the manufacturer’s instructions.

**Figure 1:**
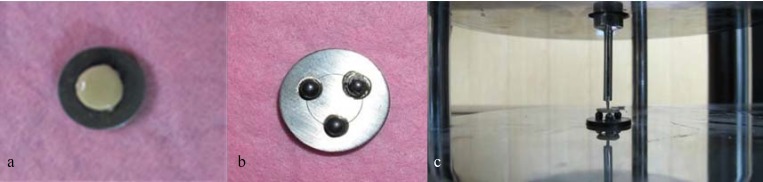
**a **Sample **b **Metal jig. **c** UTM

All specimens were fired under similar furnace conditions. After firing the specimens for a second time and polishing them with a rubber disc, the specimens reached 1.6 mm in thickness (0.4 mm for the frame thickness, 0.2 for the opaque layer thickness, and 1 mm for the dentin thickness) Figure 1a.

Finally, in order to evaluate shear bond strength of the specimens, they were fixed on a metal jig and subjected to three-point bending test [8] Figure 1b. They were then placed in the UTM (ZwickRoell; 2050, Germany) in such a way that the metal side of the specimens was in the direction of the machine’s tip and the porcelain was in the opposite side. Then, specimens were loaded in the center by the cylinder head of the machine (0.7 mm in diameter, at the speed of 0.5mm/min) Figure 1c. Shear forces were applied along the metal-porcelain interface until the first fracture appeared. The values, obtained from measuring the fracture force, were in newton (N) but they were converted to mega Pascal (MPa) through applying the following equation which is related to the three-point bending test [8, 17].

(MPa) Shear strength (t) = f⁄d.h.3.14

F= the exerted force load in Newton

D= the diameter of the punch or the machine’s tip in mm

H= the thickness of specimens in mm

## Results

The results indicated that shear bond strength of Ni-Cr-T3 alloy was higher than the other alloys (94 MPa). 

**Table 1:** Descriptive statistics of the data

**Alloys**	**N**	**Mean**	**SD**	**Minimum**	**Maximum**
Super Cast	15	2.7084E2	32.60363	207.34	322.45
Ni-cr-T3	15	3.3067E2	22.33740	297.62	365.21
VeraBond	15	2.4554E2	28.47268	171.84	286.04
X-33	15	2.3484E2	19.34222	182.96	264.17
Total	60	2.7047E2	45.32111	171.84	365.21

Super Cast alloy had the next greatest shear bond strength (80. 87 MPa). And VeraBond (69.66 MPa) and x-33 alloys (66.53 MPa) took the third place simultaneously.

The above table shows the mean and standard deviation values for shear bond strength of the four relevant alloys. One-Way analysis of variance results indicated that there was a statistically significant difference among the alloys in their shear bond strength (*p*< 0.001). The results of LSD test further showed that there was a statistically significant difference in shear bond strength between Super Cast alloy and Ni-cr-T3 and X-33 alloys (*p*< 0.001); Super Cast alloy and VeraBond (*p*< 0.05); Ni-cr-T3 alloy and VeraBond and X-33 alloys (*p*< 0.001).

However, the results showed that there was no statistically significant difference between VeraBond and X-33 alloys regarding their shear bond strength (*p*> 0.05). 

One-Way ANOVA results showed that the four alloys were mostly different from each other in their shear bond strength. Tukey test further revealed the shear bond strength of Ni-cr-T3 alloy was higher than the other three alloys. The shear bond strength of Super Cast alloy was lower than that of Ni-cr-T3 alloy but higher than the shear bond strength of VeraBond and X-33 alloys; and finally the shear bond strength of VeraBond alloy was lower than that of Super Cast alloy but VeraBond and X-33 alloys were not significantly different from each other in their shear bond strength. 

## Discussion

One of the most broadly used dental restoration procedures is the application of metal-ceramic restorations. Originally, gold alloys were used to make these restorations but with a rise in gold prices, the application of lower-cost alloys became more common. Ni-Cr alloys have a high modulus of elasticity (ME) and they bend less under extreme occlusal loads. Thus they can be considered as a good substitute for gold alloys. Despite the many advantages of metal-ceramic restorations, the increase in reports of porcelain chipping has aroused great concerns [2]. Nowadays, prosthodontists and dentists use different combinations of alloys and porcelain to achieve an optimal metal-porcelain bond and to increase success in metal-ceramic restorations. Therefore, an accurate selection of a compatible alloy and porcelain combination is of great importance [2, 5]. With regard to the facts that dental porcelain has the weakest strength in facing shear forces and that these forces are one of the main reason for porcelain chipping in the clinic, this study evaluated shear bond strength of three base-metal alloys and one noble alloy with the common widely used VMK Master Porcelain [9]. Metal-porcelain bond strength is influenced by mechanical interlocking and van der Waals forces [10]. The use of three-point bending test is a basic and simple method for evaluating shear bond strength [8]. This study benefitted from this test in order to evaluate all the specimens and, sequentially, the use of this test depends on the ME of the alloy. Based on the previous researches, the values for porcelain bond failure range from 15 to 39 MPa and metal-ceramic bond strength values are from 55-103 MPa, depending on the type of alloy [8]. Accordingly, the least shear bond strength between metal and ceramic is required for the fracture to occur in the porcelain itself rather than in the metal-porcelain interface [8]. The findings of this study are in line with the results of many studies which have investigated metal-porcelain shear bond strength and porcelain mixed-fracture (fracture of metal-ceramic adhesive bond and porcelain bonding fracture). Some problems occur in investigating metal-porcelain shear bond strength since stress concentration is different along metal-ceramic interface [8]. Furthermore, no pure shear stress can lead to a clinical fracture [8]. The findings of this study indicated that the shear bond strength between VMK Master Porcelain and Ni-cr-T3 base-metal alloy was significantly higher than the other groups. In addition, shear bond strength of Super Cast base-metal alloy was lower than that of Ni-cr-T3 base-metal alloy but higher than VeraBond base-metal and X-33 noble alloys. However, we experienced that there was no statistically significant difference between VeraBond and X-33 alloys in their shear bond strength. It should be mentioned here that the obtained mean values for shear bond strength of all four groups were clinically in the acceptable range and in line with the previous studies [8]. The overall results obtained in this study, however, are different from some of the previous studies [8] and this can be due to differences in the research methods. Although shear bond strength of X-33 noble alloy is clinically acceptable (66.53 MPa), it is lower than the other alloys. One reason for the low shear bond strength of this alloy, in comparison with the other two base-metal alloys (Super Cast and VeraBond base-metal alloys) could be due to the presence of high Pd content in this alloy. When increasing temperature, the high content of Pd can lead to rapid absorption of hydrogen. The absorbed hydrogen is released during firing the opaque layer of porcelain and this causes porosities in porcelain. The areas with porosities are not strong enough to withstand loadings, and thus; clinical fractures occur in them [13, 15]. Furthermore, sandblasting of the specimens causes more roughness in these alloys surfaces; since it has a low ME. During firing, opaque porcelain does not flow enough to penetrate into the rough surface; therefore, the porosities can be considered as an important factor which contribute to metal-porcelain debonding under the exerted forces; especially shear forces. 

Noble alloys have a low ME, therefore, they bend more than base-metal alloys under great forces such as occlusal forces and finally the frequent occurrence of bending leads to porcelain chipping overtime [8].

Ni-cr-T3 base-metal alloy, with a high ME, has the greatest shear bond strength since it does not face the aforementioned problems caused by having low ME. VeraBond had the lowest shear bond strength among the base metal alloys. It contains less chromium than the other two base-metal alloys. This may lead to the formation of a thin oxide layer which can negatively affect the initial bond between porcelain and metal [17]. 

Despite all these differences, shear bond strength of all the four alloys is greater than porcelain bond strength. Clinically, the strength value of all the four groups are in the acceptable range, thus they can be used in the mouth. It should be mentioned that this study benefitted from the use of three-point bending test. The use of this test depends on the alloy’s ME in that a high ME reduces the force in the metal-porcelain interface and increases the bond strength. However, other tests can be used in future to realize whether the same results will be obtained or not. 

## Conclusion

It can be concluded that the base-metal alloys, especially Ni-cr-T3 can serve as a good substitute for the high-cost noble alloys. Different factors affect metal-porcelain shear bond strength of which one of the most important is the adjustment between the metal and the porcelain. Therefore, time and cost are not the only determining factors in selecting the most effective alloy for metal-ceramic restorations. All the properties of the alloy and all the factors that might affect the shear bond strength should be considered. 

## References

[1] Hsu CS, Wang CC (1997). The shear bond strength of porcelain and base metal alloys for metal-ceramic crown (VI). Kaohsiung J Med Sci.

[2] do Prado RA, Panzeri H, Fernandes Neto AJ, das Neves FD, da Silva MR, Mendonça G (2005). Shear bond strength of dental porcelains to nickel-chromium alloys. Braz Dent J.

[3] Aladağ A, Cömlekoğlu ME, Dündar M, Güngör MA, Artunç C (2011). Effects of soldering and laser welding on bond strength of ceramic to metal. J Prosthet Dent.

[4] Bauer JR, Loguercio AD, Reis A, Rodrigues Filho LE (2006). Microhardness of Ni-Cr alloys under different casting conditions. Braz Oral Res.

[5] Bauer J, Costa JF, Carvalho CN, Grande RH, Loguercio AD, Reis A (2012). Characterization of two Ni-Cr dental alloys and the influence of casting mode on mechanical properties. J Prosthodont Res.

[6] Atsü S, Berksun S (2000). Bond strength of three porcelains to two forms of titanium using two firing atmospheres. J Prosthet Dent.

[7] Anusavice KJ (2012). Standardizing failure, success, and survival decisions in clinical studies of ceramic and metal-ceramic fixed dental prostheses. Dent Mater.

[8] Rosenstiel SF, Land MF, Fujimoto J (2006). Contemporary fixed prosthodontics.

[9] Sipahi C, Ozcan M (2012). Interfacial shear bond strength between different base metal alloys and five low fusing feldspathic ceramic systems. Dent Mater J.

[10] Asakura M, Kominami Y, Hayashi T, Tsuruta S, Kawai T (2012). The effect of zinc levels in a gold-based alloy on porcelain-metal bonding. Dent Mater.

[11] Vickery RC, Badinelli LA (1968). Nature of attachment forces in porcelain-gold systems. J Dent Res.

[12] Iwama H, Nakamura K (1976). Bonding strength between porcelain and gold alloys (2) The effects of iron, indium and tin (author's transl). Shika Rikogaku Zasshi.

[13] Bauer JR, Grande RH, Rodrigues-Filho LE, Pinto MM, Loguercio AD (2012). Does the casting mode influence microstructure, fracture and properties of different metal ceramic alloys?. Braz Oral Res.

[14] Shell JS, Nielsen JP (1962). Study of the bond between gold alloys and porcelain. J Dent Res.

[15] Pretti M, Hilgert E, Bottino MA, Avelar RP (2004). Evaluation of the shear bond strength of the union between two CoCr-alloys and a dental ceramic. J Appl Oral Sci.

[16] Choi BK, Han JS, Yang JH, Lee JB, Kim SH (2009). Shear bond strength of veneering porcelain to zirconia and metal cores. J Adv Prosthodont.

[17] Madhav VN, Padmanabhan TV, Subramnian R (2012). Evaluation of flexural bond strength of porcelain to used nickel-chromium alloy in various percentages. Indian J Dent Res.

